# Pathology-Informed Personalized Exoskeleton Assistance for Post-Stroke Gait Rehabilitation via Simulation-to-Real Reinforcement Learning

**DOI:** 10.3390/healthcare14111523

**Published:** 2026-05-30

**Authors:** Chuyi Ou, Yinbin Peng, Furong Zhang

**Affiliations:** 1 Department of Rehabilitation and Exercise Therapy, Chengdu University of Chinese Traditional Medicine-Keele Joint Health and Medical Institute, Chengdu 611137, China; ou.chuyi@stu.cdutcm.edu.cn; 2 School of Computer Science and Engineering, University of Electronic Science and Technology of China, Chengdu 611731, China; 3 School of Health Preservation and Rehabilitation, Chengdu University of Traditional Chinese Medicine, Chengdu 610075, China; zhangfurong@cdutcm.edu.cn

**Keywords:** stroke rehabilitation, gait analysis, exoskeleton assistance, deep reinforcement learning, transfer learning

## Abstract

**Background/Objectives:** Post-stroke gait impairment is highly heterogeneous, which limits the effectiveness of standardized exoskeleton control strategies. Deep reinforcement learning offers a route to adaptive assistance, but its use in stroke rehabilitation is constrained by limited pathological gait data and the lack of interpretable transfer frameworks. We developed a data-efficient, pathology-informed reinforcement learning framework for personalized exoskeleton assistance under limited clinical gait data. **Methods:** The framework combines neuromuscular-inspired parametric augmentation (NIPA) with parameter-efficient transfer learning. NIPA synthesizes pathological gait trajectories by modeling weakness, stiffness or contracture, and abnormal synergies. A policy is first pretrained in simulation and then adapted to clinical gait data by freezing a shared feature extractor and fine-tuning the output heads. The framework was evaluated on a public clinical gait dataset of 50 stroke survivors using tracking error, reward, smoothness, generalization, and data efficiency as main outcomes. **Results:** The proposed method outperformed zero assistance, rule-based control, and reinforcement learning from scratch on the test set. Compared with scratch, it reduced total MSE from 14.8681 to 11.9369 ($p=5.96\times 10^{-8}$) and improved reward from −21.2264 to −18.4798 ($p=3.76\times 10^{-4}$). Hip MSE decreased from 5.9544 to 4.0143 ($p=7.51\times 10^{-8}$) and knee MSE decreased from 6.5507 to 5.4507 ($p=1.51\times 10^{-5}$), with significant improvements in repeated experiments. **Conclusions:** The proposed framework reduces reliance on large pathological training datasets and improves offline trajectory-level personalization under limited clinical data. It also provides an interpretable basis for quantitative characterization of post-stroke gait heterogeneity and may support individualized rehabilitation assessment and assistance planning.

## 1. Introduction

Stroke remains a leading cause of long-term disability worldwide. Each year, about 15 million people experience stroke, and 70–80% of survivors live with persistent motor deficits that limit mobility and independence [1,2]. Gait impairment is among the most common consequences. Muscle weakness, spasticity, abnormal synergies, and reduced joint range of motion can all disrupt walking and reduce quality of life [3,4]. Rehabilitation is difficult because post-stroke gait deficits vary substantially across patients in lesion location, recovery stage, and anthropometry, so standardized treatment protocols often have limited effect [5]. In addition, recovery depends on intensive, repetitive, task-specific training, which is difficult to sustain with conventional therapy alone under typical clinical resource constraints [6].

Robotic exoskeletons have drawn increasing attention as a way to deliver intensive, repeatable, and quantifiable gait training [7,8]. Their control strategies have evolved from traditional model-based methods, such as impedance control and trajectory tracking [9], to more adaptive data-driven approaches [10]. Among these, deep reinforcement learning (DRL) is appealing because it can learn assistance policies directly from interaction data without requiring an explicit system model [11,12]. Recent studies have applied RL to lower-limb exoskeleton trajectory tracking [13], adaptive hip torque modulation [14], and personalized impedance adjustment in rehabilitation settings [15,16,17]. However, DRL remains difficult to translate to stroke rehabilitation because it typically requires large amounts of interaction data, which are hard to obtain in clinical populations for both practical and ethical reasons [12,18,19]. Online exploration also raises safety concerns because poorly trained policies may generate unstable actions [20].

Simulation-based training is one possible way to reduce the burden of real data collection. Domain randomization is widely used to narrow the gap between simulation and reality by injecting unstructured variability during training [21,22,23,24]. More explicit domain adaptation methods attempt to handle distribution shifts through feature alignment or cross-domain mapping [25]. For example, Luo et al. [26] used a CycleGAN-based framework to translate between simulated and real sensor data without paired samples. Still, standard domain randomization mainly perturbs physical parameters as noise, whereas post-stroke gait deviations reflect structured neuromuscular impairment rather than arbitrary disturbance. In addition, most existing adaptation methods in rehabilitation focus on perception tasks such as intent recognition or sensor mapping, with relatively little work on closed-loop control policies [27,28].

A related line of work uses few-shot learning or meta-learning to support rapid personalization from limited patient-specific data [29,30,31]. Many of these studies transfer knowledge from healthy populations to individual stroke survivors [29,30]. This idea is attractive, but meta-learning usually requires many diverse training tasks, which are difficult to assemble in stroke rehabilitation [32]. Moreover, much of the existing few-shot literature emphasizes state estimation rather than closed-loop control, and catastrophic forgetting remains a concern when adaptation data are extremely limited [33].

Another practical question is how to build pretraining data that reflect pathological gait structure. Generative models have become a common way to synthesize human motion [18,34,35,36]. Parameterized biomechanical models, including OpenSim-based approaches, offer a more mechanism-oriented alternative by modifying healthy templates according to physiological principles [3]. Yet many generative methods operate in sensor space and do not explicitly represent impairment mechanisms [37,38,39]. Detailed biomechanical simulation can be informative, but it is often computationally expensive and difficult to tailor to many subjects. Existing approaches also rarely produce paired healthy and pathological trajectories that are well suited for pretraining assistive control policies.

Taken together, prior work has advanced simulation-to-real adaptation [21,40], few-shot learning [17,41], and pathological gait synthesis [34,42], but these components are rarely integrated into a single framework for data-efficient post-stroke gait personalization. At the same time, post-stroke gait is not only a control problem. It also reflects underlying motor impairment. Weakness, stiffness, abnormal muscle synergies, and reduced joint excursion contribute to patient-specific movement patterns that are relevant to rehabilitation assessment and assistance design. This motivates a framework that links impairment mechanisms to kinematic deviations while remaining useful for policy adaptation. In this study, the target domain is a heterogeneous pathological gait distribution represented by clinical kinematic data rather than direct hardware interaction. We therefore propose a data-efficient simulation-to-real framework that combines pathology-informed pretraining with lightweight transfer to improve offline adaptation under limited clinical data.

The principal contributions of this work are threefold:

- Clinically Interpretable Pathological Gait Modeling: We introduce Neuromuscular-Inspired Parametric Augmentation (NIPA), a mechanism-driven method that synthesizes diverse pathological gait trajectories by explicitly modeling stroke-related impairment mechanisms, including weakness, stiffness, and abnormal synergies. Unlike unstructured perturbation strategies, NIPA preserves interpretable links between impairment mechanisms and kinematic deviations.
- Data-Efficient Simulation-to-Real Personalization: We develop a partial transfer learning strategy that preserves the pretrained feature extractor while adapting only lightweight task-specific output layers to individual patients. This design improves data efficiency under limited clinical samples and mitigates catastrophic forgetting during patient-specific adaptation.
- Quantitative Evaluation on Clinical Gait Data: We evaluate the proposed framework on a public clinical gait dataset [43]. Results show improved tracking performance, smoothness, generalization, and few-shot adaptation relative to representative baselines, while supporting quantitative analysis of heterogeneous post-stroke gait patterns.

## 2. Materials and Methods

This section presents a simulation-to-real transfer reinforcement learning framework for exoskeleton assisted stroke gait rehabilitation. The method targets data scarcity, subject heterogeneity, and the domain gap between simulation and clinical deployment. The overall pipeline consists of (i) reinforcement learning with an assist-as-needed action design that enforces a clinically interpretable safe corridor, (ii) neuromuscular inspired parametric augmentation for large scale pretraining data generation, and (iii) backbone freezing and head fine tuning for rapid adaptation with few clinical samples.

### 2.1. Overall Workflow

**Figure 1:**
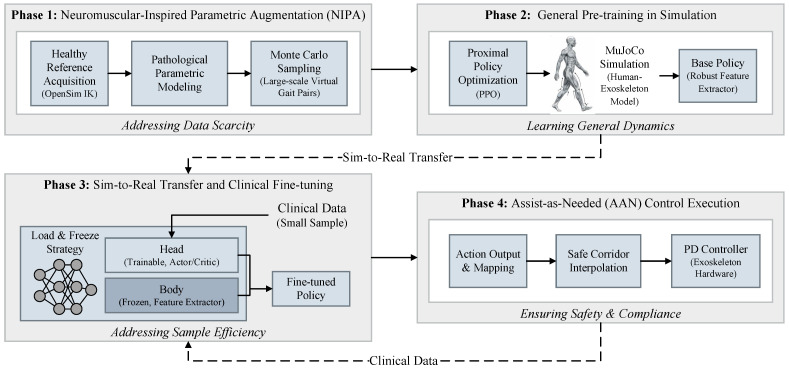
Overall workflow of the proposed simulation-to-real transfer reinforcement learning framework. Phase 1: NIPA for pathological data generation. Phase 2: General dynamics pretraining in simulation. Phase 3: simulation-to-real transfer learning. Phase 4: assist-as-needed control execution with safe corridor constraints.

We propose a simulation-to-real transfer reinforcement learning framework for exoskeleton assisted stroke gait rehabilitation. As illustrated in Figure 1, the proposed framework aims to address the challenges of clinical data scarcity and subject heterogeneity. It achieves efficient simulation-to-real transfer through four tightly coupled phases, forming a closed loop from virtual simulation to clinical deployment.

The pipeline starts with Phase 1, neuromuscular inspired parametric augmentation (NIPA). To address the tension between the data demands of deep reinforcement learning and the scarcity of clinical pathological gait data, we develop a physics-based virtual data factory. Specifically, we take standardized healthy gaits obtained from OpenSim inverse kinematics (IK) as inputs and inject clinically grounded pathological features (e.g., weakness, stiffness, and abnormal synergies) via Monte Carlo sampling. This process yields a large and diverse library of synthetic pathological gait pairs (over 2000 pairs) spanning impairment severities and provides the source domain for learning. Phase 1 is detailed in Section 2.4. Unlike conventional augmentation based on unstructured parameter perturbation, NIPA is designed to preserve clinically meaningful links between impairment mechanisms and the resulting gait deviations, making the generated source domain more suitable for both transfer learning and quantitative characterization of patient-specific pathological gait patterns.

Phase 2 performs general dynamics pretraining in a high-fidelity MuJoCo environment using Proximal Policy Optimization (PPO) [44]. This stage enables the extraction of transferable gait dynamics representations that support efficient downstream personalization. The simulation dynamics and reinforcement learning formulation used in Phase 2 are described in Section 2.2 and Section 2.3.

Phase 3 targets simulation-to-real transfer across data distributions. We adopt a simulation-to-real transfer learning strategy that reuses a pretrained feature extractor and updates only lightweight output heads with a small amount of patient data. This design reduces effective degrees of freedom, improving data efficiency while mitigating overfitting and catastrophic forgetting during adaptation. Phase 3 is described in Section 2.5.

Finally, Phase 4 executes assist-as-needed control. The adapted policy outputs bounded assistance ratios constrained by a safe corridor and realized as smooth joint position commands tracked by a low level PD controller. This execution layer is formalized in Section 2.3 and implemented within the coupled dynamics of Section 2.2.

For clarity, we first specify the coupled dynamics and the reinforcement learning formulation in Section 2.2 and Section 2.3, which are shared across phases. We then describe the phase-specific components, including NIPA (Section 2.4) and simulation-to-real transfer learning (Section 2.5).

### 2.2. Human–Exoskeleton Coupled Dynamics

We represent the human–exoskeleton interaction as a coupled multibody system in a physics-based simulator (MuJoCo), which provides the dynamical environment for policy learning. The model focuses on sagittal-plane hip, knee, and ankle motions because these joints are most directly related to clinically relevant gait deviations after stroke. Let $q\in R^{n}$ and $\dot{q}$ denote the generalized coordinates and velocities, respectively. At each simulation step, the coupled dynamics are written as

(1) $$M\left(q\right)\ddot{q}+C\left(q,\dot{q}\right)\dot{q}+g\left(q\right)=\tau +J{\left(q\right)}^{⊤}\lambda ,$$

where $\ddot{q}$ is the generalized acceleration, τ is the generalized actuation torque, and ${\left(\cdot \right)}^{⊤}$ denotes transpose. M(q) is the mass matrix. $C\left(q,\dot{q}\right)\dot{q}$ and g(q) represent Coriolis/centrifugal and gravitational terms, respectively. J(q) is the constraint Jacobian and λ denotes the corresponding Lagrange multipliers, so that $J{\left(q\right)}^{⊤}\lambda$ captures generalized forces induced by constraints such as foot ground contacts. This formulation exposes the policy to contact transitions and dynamic couplings along the gait cycle, supporting robust learning under nonlinear and hybrid dynamics. Observations are affected by modeling error, sensor noise, and inter-subject variability, and we explicitly inject these uncertainties during training via perturbations of key physical parameters and observation noise. To provide clinically interpretable targets, we use a phase-conditioned reference generator to output $q_{H}\left(t\right)$ and $q_{S}\left(t\right)$, and the high-level policy synthesizes the commanded trajectory $q_{cmd}\left(t\right)$ within a safe corridor. The low-level PD controller uses the same form in simulation and deployment,

(2) $$\tau \left(t\right)=K_{p}\left(q_{cmd}\left(t\right)-q_{A}\left(t\right)\right)-K_{d}{\dot{q}}_{A}\left(t\right),$$

where $q_{cmd}\left(t\right)$ denotes the commanded joint angle trajectory synthesized by the high-level policy within the safe corridor. *t* denotes time. $q_{A}\left(t\right)$ and ${\dot{q}}_{A}\left(t\right)$ denote the measured joint angles and velocities of the actuated joints, and $K_{p}$ and $K_{d}$ are proportional and derivative gain matrices. This controller converts the high-level assistance decision into a hardware-realizable command while preserving clinically acceptable smoothness in trajectory correction.

### 2.3. Reinforcement Learning Formulation

We formulate gait assistance as a finite-horizon Markov decision process (MDP) denoted by (S,A,P,R,γ). The high-level policy acts as the decision-making agent, and the environment includes the coupled dynamics and the reference trajectory generator. At each time step *t*, the policy outputs an action $a_{t}$, and the environment returns a reward $r_{t}$ and the next state $s_{t+1}$.

To describe the patient state in a way that is relevant to rehabilitation monitoring, the observation vector integrates gait phase, reference information, tracking error, and the previous action. The observation vector $s_{t}\in R^{17}$ encodes gait phase, reference trajectories, tracking error, and the previous action. We represent the phase by a continuous embedding $\left[\sin\left(\phi_{t}\right),\cos\left(\phi_{t}\right)\right]$ with $\phi_{t}\in \left[0,2\pi \right)$ to avoid the discontinuity at the cycle boundary. Let $q_{H}\left(t\right)\in R^{3}$ denote the healthy reference joint angles and $q_{S}\left(t\right)\in R^{3}$ denote the stroke baseline joint angles for hip, knee, and ankle. Let $q_{A}\left(t\right)\in R^{3}$ denote the current exoskeleton joint angles. We define the tracking error as $e_{track}\left(t\right)=q_{A}\left(t\right)-q_{H}\left(t\right)$. We include the previous action $a_{t-1}\in R^{3}$ to encourage temporal smoothness. The final state is

(3) $$s_{t}=\left[\sin\left(\phi_{t}\right),\cos\left(\phi_{t}\right),q_{H}\left(t\right),q_{S}\left(t\right),q_{A}\left(t\right),e_{track}\left(t\right),a_{t-1}\right].$$

From a rehabilitation monitoring perspective, this observation vector summarizes the current gait phase, deviation from the therapeutic reference, and individualized target information. The policy therefore outputs assistance commands conditioned on the current motor state and the intended rehabilitation goal.

To ensure safe and clinically interpretable assistance, we design the action as a bounded assistance ratio rather than direct torque commands. The policy outputs a raw action $a_{t}\in {\left[-1,1\right]}^{3}$, which is mapped to an assistance ratio $\beta_{t}\in {\left[0,1\right]}^{3}$ by

(4) $$\beta_{t,i}=\frac{a_{t,i}+1}{2},\;i\in \left\{\mathrm{hip},\mathrm{knee},\mathrm{ankle}\right\}.$$

We generate the commanded joint trajectory $q_{cmd}\left(t\right)$ by linear interpolation between the stroke baseline and the healthy reference,

(5) $$q_{cmd,i}\left(t\right)=q_{S,i}\left(t\right)+\beta_{t,i}\left(q_{H,i}\left(t\right)-q_{S,i}\left(t\right)\right).$$

When $\beta_{t}\to 0$, the controller behaves transparently and follows the stroke baseline. When $\beta_{t}\to 1$, the controller enforces the healthy reference for maximal correction. This interpolation defines a safe corridor because the command remains within the convex hull spanned by two clinically derived trajectories. In practical terms, the policy is encouraged to correct pathological motion without leaving a clinically interpretable range of assistance. The low-level PD controller computes torques as

(6) $$\tau \left(t\right)=K_{p}\left(q_{cmd}\left(t\right)-q_{A}\left(t\right)\right)-K_{d}{\dot{q}}_{A}\left(t\right),$$

where $K_{p}$ and $K_{d}$ are fixed stiffness and damping gains.

Having defined the action space and low-level control law, we next specify the reward function used to balance several rehabilitation-oriented objectives. The reward encourages accurate tracking, feasible command following, and smooth assistance transitions. We define the reward as

(7) $$\begin{array}{c} r_{t}=\lambda_{bound}\left(t\right)\;w_{track}\;r_{track}+w_{cmd}\;r_{cmd}+w_{smooth}\;r_{smooth},\\ r_{track}=-{∥q_{A}\left(t\right)-q_{H}\left(t\right)∥}^{2},\\ r_{cmd}=-{∥q_{A}\left(t\right)-q_{cmd}\left(t\right)∥}^{2},\\ r_{smooth}=-{∥a_{t}-a_{t-1}∥}^{2}. \end{array}$$

Here $r_{track}$, $r_{cmd}$, and $r_{smooth}$ measure tracking deviation from the healthy reference, command consistency, and abrupt action variation, respectively. The scalars $w_{track}$, $w_{cmd}$, and $w_{smooth}$ weight the corresponding terms. We introduce a phase-dependent weight $\lambda_{bound}\left(t\right)\in \left[0.1,1.0\right]$ that reduces the weight of the terminal portion of the gait cycle to mitigate instability near contact transitions and phase reset. Overall, this reward design favors assistance that is accurate enough to support trajectory correction while remaining smooth enough for rehabilitation use.

Based on the defined Markov decision process, we use Proximal Policy Optimization (PPO) to estimate the policy parameters. PPO is adopted because its clipping mechanism limits excessively large policy updates and improves training stability under heterogeneous gait dynamics. The training process follows an iterative sample–evaluate–update loop. First, the agent interacts with the environment to collect trajectory data. Second, Generalized Advantage Estimation (GAE) is used to compute the advantage function ${\hat{A}}_{t}$ and balance variance against bias. Finally, the policy parameters θ are updated by maximizing the following clipped surrogate objective:

(8) $$L^{CLIP}\left(\theta \right)={\hat{E}}_{t}\left[\min\left(\rho_{t}\left(\theta \right){\hat{A}}_{t},\mathrm{clip}\left(\rho_{t}\left(\theta \right),1-\epsilon ,1+\epsilon \right){\hat{A}}_{t}\right)\right],$$

where $\rho_{t}\left(\theta \right)=\frac{\pi_{\theta }\left(a_{t}|s_{t}\right)}{\pi_{\theta_{old}}\left(a_{t}|s_{t}\right)}$ is the probability ratio between the new and old policies, $\theta_{old}$ denotes the policy parameters before the update, and the clip(·) operation constrains $\rho_{t}\left(\theta \right)$ within [1−ϵ,1+ϵ] (typically ϵ=0.2) to improve update stability. For the present application, PPO offers a practical compromise between optimization stability and data efficiency, both of which are important when adapting assistance policies to clinically heterogeneous subjects.

### 2.4. Neuromuscular Inspired Parametric Augmentation

Clinical stroke gait data are scarce and heterogeneous, which limits direct learning of individualized assistance policies. We propose NIPA to generate large-scale synthetic pathological gait trajectories for simulation pretraining. NIPA takes standardized healthy gait trajectories as inputs and applies parameterized pathology operators with Monte Carlo sampling to produce paired pathological trajectories. The quantitative and visual validation of the generated pathological trajectories is reported in Section 3.1.

NIPA injects clinically interpretable neuromuscular impairment mechanisms into healthy gait trajectories through a set of parameterized operators. Given an input joint trajectory $q_{in}\left(t\right)$, we consider three impairment categories: weakness, stiffness and contracture, and abnormal synergies. The weakness operator scales the trajectory amplitude,

(9) $$q_{weak}\left(t\right)=q_{in}\left(t\right)\left(1-\lambda_{weak}\right).$$

The stiffness and contracture operator compresses the range of motion around a nominal center $\bar{q}$ and adds a bias,

(10) $$q_{stiff}\left(t\right)=\bar{q}+\left(q_{in}\left(t\right)-\bar{q}\right)\left(1-\lambda_{stiff}\right)+\delta_{bias}.$$

where $\bar{q}$ denotes a nominal joint angle center and $\delta_{bias}$ is a constant offset capturing contracture induced shifts. The abnormal synergy operator couples a target joint toward a fixed pathological posture $q_{fixed}$ with a time varying weight,

(11) $$q_{coupled}\left(t\right)=\left(1-\omega_{syn}\left(t\right)\right)q_{in}\left(t\right)+\omega_{syn}\left(t\right)q_{fixed}.$$

where $q_{fixed}$ denotes a fixed pathological posture for the target joint. We set $\omega_{syn}\left(t\right)=\lambda_{syn}\;\sigma \;\left(q_{source}\left(t\right)\right)$, where $q_{source}\left(t\right)$ is a source joint trajectory and σ(·) is a bounded activation function. The operators can be composed sequentially to simulate compound impairments, with $q_{in}\left(t\right)$ denoting the stage wise input trajectory.

Using Monte Carlo sampling, we construct a synthetic dataset spanning diverse impairment types and severities. Healthy reference curves $q_{H}\left(t\right)$ are obtained from OpenSim inverse kinematics (IK) using the generic Gait2354 model and marker trajectories from standard clinical motion capture. We apply small random amplitude scaling (±10%) and phase shifts to form $q_{H}^{\prime }\left(t\right)$, sample impairment parameters under clinically guided priors, and then apply the transformations sequentially from local to global,

(12) $$q_{sim}=T_{syn}\;\left(T_{stiff}\;\left(T_{weak}\left(q_{H}^{\prime }\right)\right)\right),$$

where $q_{H}^{\prime }$ denotes the base augmented healthy reference trajectory, serving as the basis for pathological transformation. Here $T_{weak}$, $T_{stiff}$, and $T_{syn}$ denote the weakness, stiffness/contracture, and synergy operators, respectively, with $T_{syn}$ applied last. The resulting paired data $\left(q_{H},q_{sim}\right)$ constitute the source domain for simulation pretraining. Table 1 summarizes the clinically guided sampling configuration used by NIPA across joints and impairment types. The probability (Prob.) specifies the activation frequency of each impairment, and the range specifies a uniform prior over severity parameters. Here $\lambda_{weak}$, $\lambda_{stiff}$, and $\lambda_{syn}$ control the magnitudes of weakness, stiffness/contracture, and synergy perturbations, respectively, and their settings are chosen based on clinical experience to span severities from mild deficits (e.g., slight drop foot) to severe patterns (e.g., extensor synergy). In this sense, the interpretability of NIPA arises from its parameterized impairment operators: the type, joint location, and severity of simulated deficits can be explicitly controlled rather than treated as unstructured noise. Such parameter control may allow clinicians or researchers to generate patient-relevant pathological gait samples for source-domain pretraining, thereby reducing the amount of patient-specific data and adaptation time required for a new user.

This probabilistic design yields both isolated impairments and compound patterns, with parameter ranges spanning severities from mild to severe. Using this pipeline, we generated more than 2000 trajectory pairs with diverse impairment types and severities. The resulting dataset broadens the coverage of pathological gait patterns relative to available clinical data and supports robust policy pretraining.

### 2.5. Simulation-to-Real Transfer Learning

**Figure 2:**
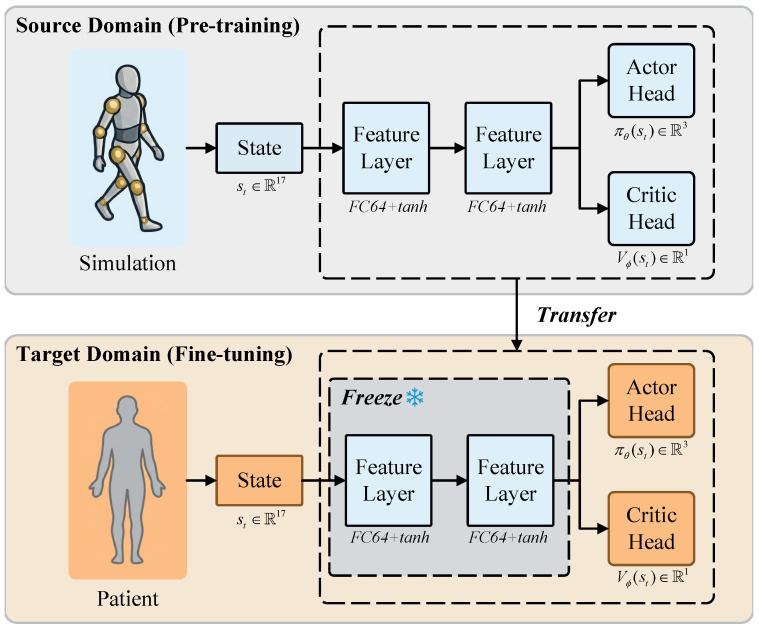
Schematic of the simulation-to-real transfer framework. The architecture comprises a shared feature extractor (Body) and task-specific heads (Actor and Critic). During target domain adaptation, the feature extractor parameters are frozen to retain generic gait dynamics learned from simulation, while the heads are fine tuned to accommodate subject-specific pathological deviations.

To adapt the pretrained policy to clinical data with limited samples, we employ a simulation-to-real transfer strategy (Figure 2). The central assumption is that simulation pretraining mainly captures subject-independent gait regularities in the shared representation, whereas subject-specific deviations are more likely to appear in the output mapping. We therefore freeze the shared feature extractor and fine-tune only lightweight output heads, which reduces the number of trainable parameters and helps preserve the clinically useful structure learned during pretraining.

The concrete implementation relies on an actor–critic architecture with a shared body and two output heads, together with a staged fine-tuning protocol. Given an observation $s_{t}$, the shared feature extractor maps it to a latent representation $z_{t}=f_{\psi }\left(s_{t}\right)$, from which the actor and critic estimate the action and state value, respectively. The feature extractor is a two-layer multilayer perceptron with 64 units per layer and tanh activations, yielding $z_{t}\in R^{64}$. The actor head implements $a_{t}=\tanh\left(g_{\theta }\left(z_{t}\right)\right)$ and outputs a three-dimensional continuous action corresponding to the assistive coefficients for the hip, knee, and ankle joints. The critic head implements $V_{\omega }\left(s_{t}\right)=h_{\omega }\left(z_{t}\right)$ and outputs a scalar state value to stabilize advantage estimation and policy optimization. In Stage 1, we pretrain the full parameter set (ψ,θ,ω) in simulation using the large-scale NIPA-generated dataset until convergence. In Stage 2, we initialize the clinical-domain policy with the pretrained parameters and freeze ψ so that gradients no longer update the shared body. In Stage 3, we continue optimization on the limited clinical samples by updating only the actor and critic heads (θ,ω). This staged design supports rapid subject-specific adaptation while preserving the more general gait structure learned in simulation, which is particularly useful when fewer than 30 subjects are available.

### 2.6. Clinical Dataset, Baselines, and Training Setup

We utilize the Van Criekinge et al. [43] clinical dataset, which comprises lower-limb kinematics from 138 able-bodied individuals and 50 stroke survivors. For each of the 50 stroke patients, we randomly assign a healthy subject as the rehabilitation target. This random healthy–stroke pairing is used only to construct a standardized computational target for controlled offline comparison and should not be interpreted as a patient-specific clinical prescription. In real rehabilitation practice, the target trajectory should be selected or adapted by clinicians according to patient ability, recovery stage, comfort, and safety constraints. These 50 paired datasets are randomly split into 25 training pairs and 25 test pairs; the training set is used for training the Scratch baseline and fine tuning.

The MuJoCo-based platform uses the Gait2354 musculoskeletal model (23 DOFs). We emulate the real domain by injecting patient kinematics as tracking targets. The hierarchical AAN control architecture outputs assistance ratios $\alpha \in {\left[0,1\right]}^{3}$ from the high-level policy, which interpolates between stroke and healthy baselines to generate commands $\theta_{cmd}$ tracked by a low-level PD controller ($K_{p}=120,K_{d}=8$).

We evaluate the proposed method, denoted as Ours, against five baselines to assess kinematic accuracy, assistance efficiency, smoothness, and the relative contribution of model-based and domain-adaptation alternatives. These baselines span from passive assistance and heuristic control to learning-based, model-based, and feature-alignment adaptation methods:

- Zero Assistance (Zero): Simulates a transparent or disabled exoskeleton (action a=−1). It serves as a lower bound to quantify the patient’s raw performance and calculate improvement gains.
- Phase-based Heuristic Rule (Rule): Adjusts assistance based on gait phase: low (0.1) during stance and high (1.0) during swing. It represents traditional heuristic control and provides a reference for adaptive methods.
- Standard RL from Scratch (Scratch): Trains PPO directly on the target task without pretraining. It serves as a reference for the gains associated with the transfer learning strategy.
- Bounded PD Tracking (PD-B): Uses a conventional proportional-derivative tracking controller to follow the bounded command trajectory defined within the corridor between the stroke baseline and the healthy reference. This baseline does not use reinforcement learning or source-domain pretraining and provides a stronger model-based control comparator than Zero and Rule.
- Feature-Alignment Adaptation (FeatAlign): Uses the same source-domain pretraining setting as Ours but adds a feature-distribution alignment objective during target-domain adaptation to reduce the discrepancy between source and target latent representations. This baseline represents an explicit domain-adaptation strategy for evaluating whether pathology-informed pretraining with frozen feature extraction offers advantages beyond generic feature alignment.

We train policies using PPO with hyperparameters detailed in Table 2. Fine-tuning uses a lower learning rate ($10^{-4}$) and a narrower clip range (0.1) than Scratch to reduce forgetting. Both use a batch size of 128 and γ=0.995. We set entropy coef. to 0.01 and horizon to 2048 to encourage exploration and capture long-term dependencies.

### 2.7. Statistical Analysis

For all methods, experiments were repeated across five independent runs. In each run, the 50 stroke–healthy pairs were randomly re-partitioned into 25 training pairs and 25 test pairs, and the corresponding models were trained and evaluated on that split. This repeated random-split protocol was used to reduce the influence of chance results arising from any single partition of the limited cohort and to provide a more robust within-dataset evaluation. Unless otherwise stated, continuous variables are reported as mean ± standard deviation. For the compact repeated-results comparison, statistical significance was assessed on subject-level paired results using two-sided Wilcoxon signed-rank tests. The reported *p* values correspond to comparisons between Ours and Scratch for the same subjects and metrics within the repeated split protocol. Rule and Zero were retained as reference baselines in the descriptive comparison, but no additional pairwise *p* values are reported for them in the current compact table. A value of p<0.05 was considered statistically significant. These analyses were intended to provide a focused statistical comparison of the learning-based methods while preserving descriptive comparisons with heuristic and zero-assistance baselines.

### 2.8. Evaluation Protocol and Metrics

We evaluate the method from both control and rehabilitation perspectives. Error and reward provide the most direct indicators, corresponding to tracking deviation and task-objective attainment, respectively. To measure gait tracking accuracy, we report the total mean squared error (Total MSE). This metric directly reflects the similarity to the healthy reference gait and the tracking precision, and lower values are better (↓).

(13) $$\mathrm{MSE}=\frac{1}{T\cdot N}\sum_{t=1}^{T}\sum_{i=1}^{N}{\left(q_{i}\left(t\right)-q_{\mathrm{ref},i}\left(t\right)\right)}^{2}.$$

Here, $q_{i}\left(t\right)$ and $q_{\mathrm{ref},i}\left(t\right)$ denote the actual and healthy reference joint angles, and *N* and *T* denote the numbers of joints and time steps. To summarize overall policy performance, we report the return $R=\sum_{t=1}^{T}r_{t}$, where $r_{t}$ is defined in Equation (7) (higher is better, ↑). To assess output smoothness and user comfort, we report jerk (Jerk). Lower jerk indicates smoother action variations, which reduces impact and improves interaction stability, and lower values are better (↓).

(14) $$\mathrm{Jerk}=\frac{1}{T-1}\sum_{t=2}^{T}{∥a_{t}-a_{t-1}∥}^{2}.$$

Here, $a_{t}$ denotes the action vector output by the policy at time step *t*.

## 3. Results

This section reports the results from both technical and rehabilitation-oriented perspectives. We focus on four complementary aspects: validation of the generated pathological trajectories, overall assistance effectiveness, subject-level trajectory behavior, and robustness across heterogeneous pathological gait patterns.

### 3.1. Quantitative Validation of NIPA-Generated Pathological Trajectories

We compared NIPA-generated pathological trajectories with real clinical stroke gait data before using them for policy pretraining. The base healthy trajectories used by NIPA were sampled from the musculoskeletal model in the MuJoCo environment and perturbed before applying the pathology operators. To reduce the influence of sample-size imbalance, bootstrap resampling repeatedly compared the real stroke cohort with equally sized subsets sampled from the larger NIPA-generated dataset. Table 3 reports two summary metrics: peak-to-peak range, which describes the movement amplitude within one gait cycle, and mean joint angle, which describes the global angular level of each trajectory. At the whole-trajectory level, the overall peak-to-peak ranges were close between NIPA and real stroke data, with 33.254° [33.109, 33.415] for NIPA and 33.265° [30.978, 35.548] for the real stroke data. At the joint-specific level, the confidence intervals of the peak-to-peak ranges indicate a degree of similarity, whereas the mean values still differ, suggesting that these scalar metrics should be interpreted together with trajectory-level morphology. The overall mean angle was higher for NIPA, 18.467° [18.345, 18.585], than for the real stroke data, 15.448° [14.151, 16.801]. This difference is expected because the healthy trajectories sampled from the MuJoCo musculoskeletal model already have higher mean joint angles than the healthy trajectories in the Van Criekinge dataset.

**Figure 3:**
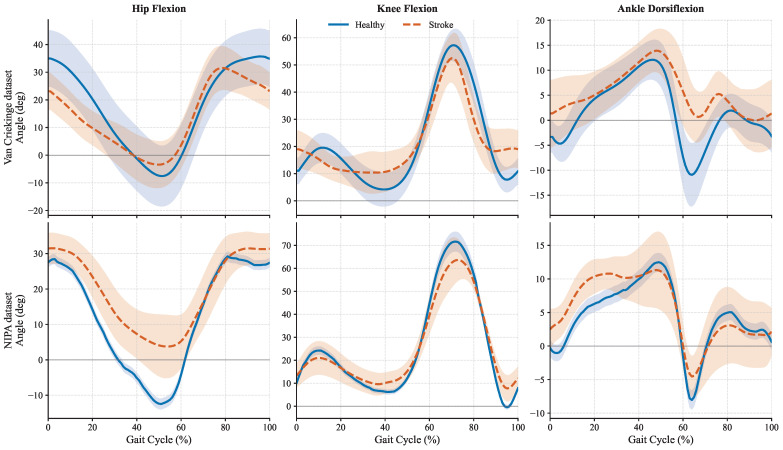
Trajectory-level comparison of joint-angle patterns between NIPA-generated data and real clinical data from the Van Criekinge dataset. Columns show hip flexion, knee flexion, and ankle dorsiflexion. Solid lines indicate healthy controls, dashed lines indicate stroke survivors, and shaded areas represent standard deviation. The top row shows real clinical data, and the bottom row shows NIPA-generated data.

Although Table 3 quantifies global angle level and amplitude scale, these scalar metrics cannot fully describe trajectory shape and phase-dependent patterns. We therefore further analyzed the trajectory means, temporal envelopes, and distribution ranges in Figure 3.

Figure 3 further shows clear overlap in the temporal envelopes and trajectory ranges of NIPA-generated and real stroke curves. This supports distributional similarity at the gait-cycle morphology level, even though the scalar means are not identical. Together, the table and figure suggest that NIPA provides a realistic overall amplitude scale, plausible gait-cycle morphology, and sufficient diversity for source-domain pretraining.

### 3.2. Quantitative Evaluation of Patient-Specific Assistance Performance

We further compare our method against five baseline controllers: a learning-from-scratch policy (Scratch), a rule-based AAN controller (Rule), a zero-assistance setting (Zero), a bounded proportional-derivative tracking controller (PD-B), and a domain-adaptation baseline based on feature distribution alignment (FeatAlign). PD-B uses a conventional bounded PD tracking law without reinforcement learning, providing a stronger model-based control baseline than Zero and Rule. FeatAlign uses the same source-domain pretraining setting as our method but aligns source and target latent feature distributions during adaptation, representing an advanced domain-adaptation baseline. Table 4 reports the subject-level tracking error and cumulative reward results on the test dataset. Across these samples, the proposed method achieves the best mean MSE and reward among the six methods, reducing the average MSE from 20.58 for Scratch, 20.77 for PD-B, and 19.02 for FeatAlign to 17.65, while improving the average reward from −18.98, −24.05, and −19.15 to −11.23.

**Figure 4:**
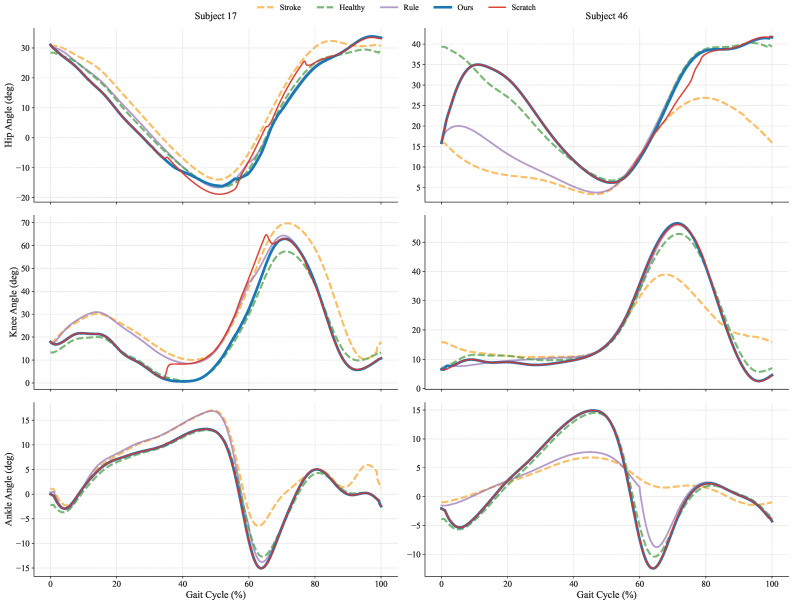
Subject-level comparison of joint trajectory tracking for Sub17 and Sub46. The plots illustrate how different control strategies modify stroke baseline gait toward the healthy reference.

To further visually demonstrate the control performance of different strategies on specific subjects, we selected two representative subjects from the test set (Sub17 and Sub46) for trajectory tracking comparison, as shown in Figure 4.

As illustrated in Figure 4, the reinforcement learning methods, namely the proposed method and the Scratch baseline, track the reference trajectories more closely than the Rule controller shown by the purple solid line. Compared with Scratch, the proposed policy shown by the blue solid line produces smoother trajectories. For Subject 17 (left column), the Scratch policy exhibits noticeable sharp peaks and oscillations in the hip and knee trajectories, particularly between 30% and 70% of the gait cycle. For Subject 46 (right column), who presents a typical stiff-gait pattern with limited knee flexion, both RL policies restore the flexion peak to approximately 55°, whereas the Scratch policy shows visible jitter in the hip trajectory during the 60% to 80% phase. In contrast, the proposed policy generates smoother trajectories that remain closer to the healthy reference shown by the green dashed line for both subjects. These subject-level examples indicate that the proposed framework can reduce unstable corrections while preserving the main gait morphology in subjects with different movement patterns. This qualitative comparison is consistent with the quantitative results.

### 3.3. Statistical Analysis of Repeated Experiments

To complement the descriptive comparison in Table 4, Table 5 summarizes repeated subject-aligned statistics for the main outcome metrics.

The repeated comparison confirms that Ours improves the main tracking and reward outcomes relative to Scratch under subject-aligned evaluation. Total MSE decreases from 14.8681 to 11.9369 (*p* = 5.96 × 10^−8^), and reward improves from −21.2264 to −18.4798 (*p* = 3.76 × 10^−4^). Hip MSE decreases from 5.9544 to 4.0143 (*p* = 7.51 × 10^−8^), and knee MSE decreases from 6.5507 to 5.4507 (*p* = 1.51 × 10^−5^), whereas the ankle difference is not significant (p=0.9578). Compared with the two stronger added baselines, Ours achieves lower total MSE than PD-B (20.2420) and FeatAlign (19.3614), and also yields higher reward than PD-B (−24.3079) and FeatAlign (−21.8160). Jerk is lower for Ours than for Scratch (0.0051 vs. 0.0069), although the rule-based and zero-assistance baselines have lower jerk because they generate much less active correction.

### 3.4. Ablation Study of Pathology-Informed Pretraining and Freezing Strategy

To disentangle the contributions of pathology-informed augmentation, source-domain pretraining, and the freezing strategy during patient-specific adaptation, we conducted a five-group ablation study. As summarized in Table 6, No Pretraining corresponds to learning directly from target-subject data without source-domain pretraining, Random + Full FT and Random + Frozen FE use non-pathology-informed random augmentation with full fine-tuning or frozen feature extraction, respectively, and NIPA + Full FT removes the freezing strategy from the proposed pathology-informed pretraining pipeline. The complete method, NIPA + Frozen FE, combines NIPA-based pretraining with frozen feature extraction during fine-tuning.

The ablation results show that the complete NIPA + Frozen FE configuration achieves the lowest Total MSE (11.9369) and the highest reward (−18.4798) among the five variants. Compared with No Pretraining, the complete method reduces Total MSE from 14.8681 to 11.9369 and improves reward from −21.2264 to −18.4798, confirming the value of source-domain pretraining for limited target-subject adaptation. Replacing random augmentation with NIPA under full fine-tuning decreases Total MSE from 14.3065 to 12.8463 and improves reward from −21.4454 to −20.3965, indicating that pathology-informed augmentation provides more useful transferable representations than generic random perturbations. Adding the freezing strategy to NIPA further improves Total MSE from 12.8463 to 11.9369 and reward from −20.3965 to −18.4798, suggesting that freezing the pretrained feature extractor helps preserve pathology-informed representations during patient-specific fine-tuning. The complete method also yields lower jerk than No Pretraining (0.0051 vs. 0.0069), indicating that the tracking improvement is not achieved at the cost of less smooth action output.

### 3.5. Robustness Across Unseen Pathological Gait Patterns

**Figure 5:**
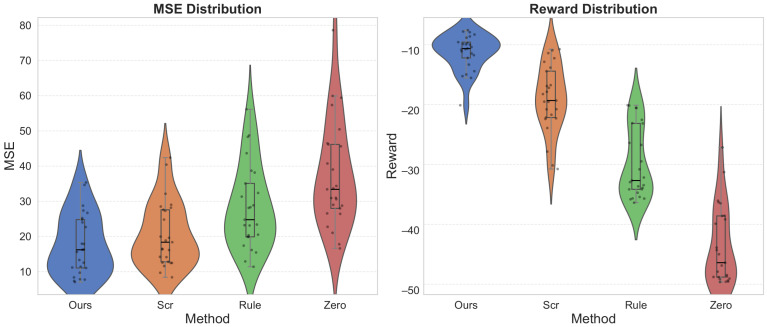
Distribution of tracking MSE and cumulative reward across 25 unseen test subjects. Narrower and lower-centered distributions indicate more stable patient-specific adaptation across heterogeneous pathological gait patterns.

This section assesses the model’s adaptability to novel patient profiles. Because the test subjects were excluded from training, their gait patterns were unseen during model fitting. Figure 5 illustrates the distribution of tracking MSE and reward for the proposed method and the Scratch, Rule, and Zero baselines across the 25 test subjects. The violin plots show that the proposed method shown by the blue violin has a more concentrated distribution in the lower MSE region (mean ≈18) than the baseline approaches. In contrast, the Scratch method (orange violin) exhibits a longer tail and higher variance. For reward, the proposed method remains concentrated around -10, whereas the Rule and Zero strategies display broader distributions with lower medians. The narrower bandwidth of the blue violin indicates lower inter-subject variability. Overall, the distributional comparison suggests more consistent performance of the proposed method across heterogeneous pathological gait patterns in this test set.

### 3.6. Stability of Patient-Specific Adaptation During Training

**Figure 6:**
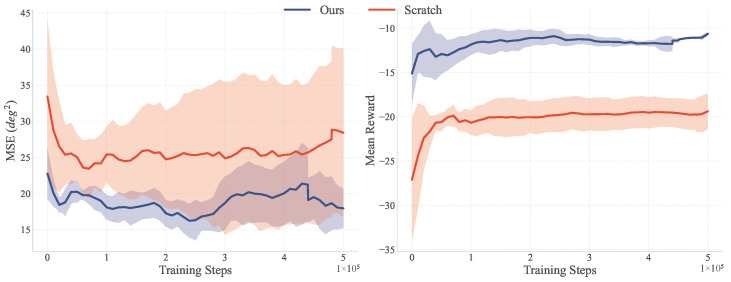
Training stability of patient-specific adaptation for Ours and Scratch over 500,000 steps. Faster convergence and narrower shaded regions indicate more stable optimization under limited clinical data.

Figure 6 illustrates the learning curves of average reward and tracking mean squared error (MSE) for both the Scratch baseline and the proposed method over 500,000 training steps. The proposed method shown by the blue curve starts with a lower MSE and a higher reward than the Scratch baseline. Throughout fine-tuning, the proposed method converges faster and shows lower variance in the later stages, as indicated by the narrower blue shaded area. In contrast, the Scratch method (red curve) improves more slowly and retains a larger performance gap, together with wider variance. Overall, the comparison indicates faster convergence and lower variance for the proposed method under the same training budget.

### 3.7. Performance Under Limited Clinical Data

**Figure 7:**
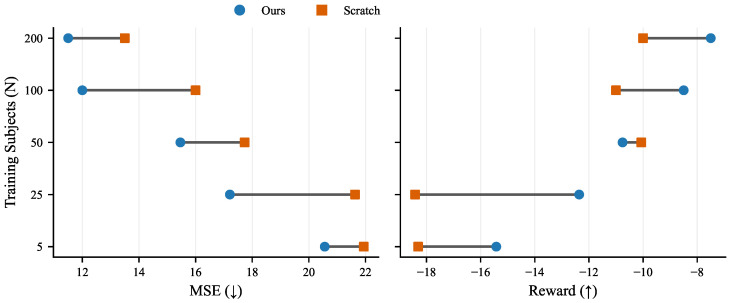
Performance under different amounts of clinical training data. The plot compares tracking MSE and cumulative reward for Ours and Scratch, highlighting the advantage of the proposed framework in low-data personalization settings.

This section evaluates performance under severe data scarcity. To assess adaptation across few-shot settings, we leveraged NIPA to augment the original training set and constructed sub-datasets with sizes N∈{5,25,50,100,200}. Figure 7 uses a dumbbell plot to visualize the performance gap between the proposed method and the Scratch baseline across these dataset sizes. The left panel shows tracking MSE (lower is better), where the proposed method (blue circles) yields lower errors than Scratch (orange squares) for all *N*. Even at N=200, the proposed method maintains lower MSE (approximately 11.5 vs. 13.5), with the largest gap appearing at N=100 (approximately 12 vs. 16). The right panel shows total reward (higher is better), where the proposed method also remains higher across the tested dataset sizes. In the low-data setting (N=25), reward is approximately −12.5 for the proposed method and approximately −18 for Scratch. At N=200, the corresponding values are approximately −7.5 and −10. These results indicate that the proposed framework retains an advantage over Scratch across the tested data regimes.

## 4. Discussion

The present results show that the proposed framework improves trajectory-level adaptation under limited clinical data conditions, with gains in tracking accuracy, convergence behavior, generalization, and data efficiency. Compared with the Scratch, Rule, and Zero baselines, the proposed method achieves a more favorable balance between assistance effectiveness and control smoothness. This pattern indicates that pathology-informed pretraining can provide a stronger initialization for personalization than learning directly from a small amount of clinical data. By exposing the policy to diverse pathological gait patterns synthesized by NIPA, the model acquires useful prior structure before adaptation to individual subjects. This may explain why the proposed method outperforms learning from scratch in tracking performance, reward, and convergence behavior. The faster convergence and lower variance observed during training are also consistent with a more stable adaptation process when subject-specific data are scarce.

A strength of the present framework is that the source-domain construction is mechanism-linked rather than purely statistical. The pathological trajectories generated by NIPA are associated with recognizable impairment mechanisms, such as weakness, stiffness, and abnormal synergies, which are commonly observed in post-stroke gait. As a result, the framework may support both transfer learning and quantitative description of patient-specific gait deviations. If future studies link these quantitative representations to established clinical scales or therapist-rated outcomes, the framework may also become more useful for clinical interpretation.

This interpretation is broadly consistent with prior studies in rehabilitation robotics, which have shown that patient-adaptive control generally outperforms fixed-rule assistance when gait patterns vary substantially across individuals [4,6,15]. At the same time, most existing approaches still rely heavily on direct subject-specific tuning or large amounts of task-specific data [26,29,45]. In this context, the present results extend previous research by suggesting that mechanism-informed pretraining can reduce the burden of adaptation while preserving subject-specific responsiveness in low-data clinical settings.

The results on unseen subjects indicate that the learned model captures cross-subject regularities in pathological gait rather than merely memorizing the training set. This matters for personalized rehabilitation because a stronger starting point can reduce the amount of calibration data needed for a new user. The few-shot experiments support this view by showing that the advantage of the proposed method becomes more pronounced in low-data settings. Together, these results indicate that simulation-to-real transfer with mechanism-informed augmentation is a useful computational approach for personalized gait assistance. In practical rehabilitation workflows, reducing the calibration burden for a new patient may improve the feasibility of individualized assistance when data collection time is limited. Likewise, more stable adaptation may facilitate translation from algorithm development to clinically usable control pipelines.

From a clinical interpretation perspective, the present outcomes should be understood as trajectory-level surrogate measures rather than direct evidence of therapeutic efficacy. Within the scope of this simulation-based study, these measures remain informative for evaluating tracking quality, overall control effectiveness, and action smoothness. The bounded assistance corridor provides an interpretable command range between the stroke baseline and the healthy reference, but it does not replace established clinical endpoints such as gait speed, Fugl–Meyer Assessment, Functional Ambulation Category, Berg Balance Scale, or therapist-rated gait quality. Therefore, the present results support trajectory-level methodological conclusions, whereas future clinical studies are still required to determine whether these improvements translate into functional rehabilitation benefits.

The joint-specific results also reveal an important limitation. Although hip and knee MSE improved significantly, the ankle difference was not significant in the repeated analysis. This result may be partly explained by the fact that both Ours and Scratch already showed relatively small ankle tracking errors, leaving limited room for further improvement. In other words, ankle assistance appears to be a relatively easier subtask in the present dataset, so the advantage of pathology-informed pretraining is less pronounced than for the hip and knee. Therefore, the proposed method should be interpreted as providing joint-dependent and non-uniform improvements rather than uniformly improving all joints.

Several limitations should be noted. First, the current study is based on offline kinematic datasets and simulation-based evaluation. Its main implication for real-time control and hardware implementation is therefore at the algorithm-design level rather than at the deployment-validation level. At the same time, the evaluation is not a purely abstract numerical test, because the proposed framework is studied in a high-fidelity simulation platform that combines a musculoskeletal gait model, exoskeleton mechanical structure, coupled control dynamics, and clinical kinematic data. This provides a meaningful intermediate level of validation for examining personalization, control adaptation, and trajectory-level assistance generation under clinically grounded movement patterns, although it does not replace direct hardware experiments. More specifically, the proposed pathology-informed pretraining strategy provides a data-efficient way to initialize patient-specific controllers under limited clinical data by exposing the model to mechanism-linked pathological gait variations before adaptation. Within this framework, assist-as-needed control should not be understood as replay of a fixed tracking trajectory, because the learned policy adjusts the bounded assistance level according to the patient state. This is relevant for future real-time exoskeleton systems because reducing the amount of subject-specific data and calibration needed before controller personalization may improve practical deployability in clinical settings. However, the present results still support conclusions only about trajectory-level adaptation and data efficiency, not about real-time human–robot interaction, hardware safety, or clinical efficacy. Second, the assistance policy is defined through a position-level bounded corridor. Although this design improves command regularity, it does not fully represent compliant physical interaction and should not be interpreted as a complete safety validation. Third, the healthy reference trajectory assigned to each stroke subject is used here as a computational target for controlled comparison, but it does not replace patient-specific therapeutic prescription in clinical practice. The present framework should also not be interpreted as a standalone clinical assessment tool in the strict clinical sense. Our study does not perform disease classification, severity grading, or clinician-validated diagnostic decision support. Instead, its main value lies in providing an interpretable computational framework for quantitative characterization of post-stroke gait heterogeneity and for supporting patient-specific rehabilitation assistance under limited data. Future work should examine how the proposed impairment-informed representation relates to clinical scales, therapist judgment, and prospective rehabilitation outcomes. Such validation will be important for determining whether the present quantitative descriptors can support clinically interpretable stratification of gait impairment and more targeted assistance planning.

Fourth, this study used repeated within-dataset splits but did not include external validation cohorts or longitudinal temporal validation. Thus, the term generalization in this work should be interpreted as within-dataset generalization to unseen subjects rather than broad clinical generalizability. Although repeated random splits were used to reduce the influence of chance results from any single partition of a limited cohort, this strategy still cannot substitute for validation on independent external datasets or longitudinal follow-up data.

Future work should extend this framework toward hardware-in-the-loop and prospective experimental validation. A natural next step is to deploy the learned policy on a physical exoskeleton platform and evaluate real time control performance, interaction comfort, and patient acceptance. It would also be valuable to incorporate multimodal measurements, such as surface electromyography and interaction forces, to improve state representation and support more responsive assistance. Further studies should investigate clinically grounded target selection and subject stratification strategies to improve translational relevance.

## 5. Conclusions

This study presents a pathology-informed and data-efficient simulation-to-real reinforcement learning framework for personalized exoskeleton-assisted gait rehabilitation after stroke. By combining neuromuscular-inspired parametric augmentation with parameter-efficient fine-tuning, the proposed method supports policy adaptation under limited clinical data while accounting for inter-subject pathological variability. Results on a public clinical gait dataset show improved tracking performance, enhanced smoothness, stronger data efficiency, and better generalization than representative baseline methods.

Beyond improved control performance, the proposed framework offers an interpretable computational basis for quantitative characterization of heterogeneous post-stroke gait patterns. This property may support individualized rehabilitation assessment and assistance planning in future exoskeleton-assisted rehabilitation systems.

## Figures and Tables

**Table 1:** NIPA Pathological Parameter Sampling Configuration.

Joint	Impairment	Operator	Prob.	Range	Clinical Meaning
Ankle	Drop Foot	Weakness	0.6	$\lambda_{weak}∼U\left(0.2,0.6\right)$	Tibialis anterior weakness, insufficient dorsiflexion
	PF Contracture	Stiffness	1.0	$\lambda_{stiff}∼U\left(0,1\right)$	Achilles tendon contracture, dorsiflexion shift
	Push-off Deficit	Weakness	0.7	$\lambda_{weak}∼U\left(0.2,0.6\right)$	Plantarflexor weakness, reduced propulsion
Knee	Hyperextension	Weakness	0.3	$\lambda_{weak}∼U\left(0.2,0.6\right)$	Knee hyperextension during stance
	Stiffness	Stiffness	0.9	$\lambda_{stiff}∼U\left(0.3,0.8\right)$	Reduced range of motion, stiff gait
Hip	Flexion Deficit	Weakness	0.3	$\lambda_{weak}∼U\left(0.2,0.6\right)$	Hip flexor weakness, reduced step length
	Extension Deficit	Stiffness	0.8	$\lambda_{stiff}∼U\left(0,1\right)$	Hip flexion contracture, limited extension
Knee & Ankle	Extensor Synergy	Synergy	0.3	$\lambda_{syn}∼U\left(0.4,0.9\right)$	Hip extension triggers knee extension/ankle PF

**Table 2:** Hyperparameter Settings for PPO Training.

Parameter	Scratch	Finetune
Total Timesteps	500,000	500,000
Learning Rate	$3\times 10^{-4}$	$1\times 10^{-4}$
Batch Size	128	128
Discount Factor (Gamma)	0.995	0.995
Clip Range	0.2	0.1
GAE Lambda	0.95	0.95
Entropy Coef	0.01	0.01
N_steps	2048	2048

**Table 3:** Bootstrap-based quantitative validation of NIPA-generated stroke trajectories against real clinical stroke trajectories. Values are reported as mean [95% confidence interval] in degrees. Peak-to-peak denotes the range of motion within a gait cycle, and mean denotes the average joint angle over the gait cycle.

Metric	Dataset	Hip	Knee	Ankle	Overall
Peak-to-peak range	NIPA stroke	30.096 [29.829, 30.360]	51.089 [50.756, 51.474]	18.576 [18.433, 18.726]	33.254 [33.109, 33.415]
	Real stroke	35.634 [33.259, 37.888]	44.684 [41.597, 47.685]	19.476 [17.758, 21.755]	33.265 [30.978, 35.548]
Mean joint angle	NIPA stroke	18.046 [17.838, 18.235]	29.380 [29.172, 29.596]	7.975 [7.807, 8.117]	18.467 [18.345, 18.585]
	Real stroke	15.582 [13.740, 17.864]	24.314 [22.449, 25.933]	6.448 [5.278, 7.664]	15.448 [14.151, 16.801]

**Table 4:** Quantitative comparison of six control strategies on the test dataset using tracking error and cumulative reward. PD-B denotes the bounded proportional-derivative tracking baseline, and FeatAlign denotes the feature-alignment domain-adaptation baseline. The best values are highlighted in bold, and the mean values across all subjects are listed in the final row. The symbols ↓ and ↑ indicate that lower and higher values are better, respectively.

Subject	MSE (↓)						Reward (↑)					
	Ours	Scratch	Rule	Zero	PD-B	FeatAlign	Ours	Scratch	Rule	Zero	PD-B	FeatAlign
Sub16	**7.38**	12.47	15.46	21.04	13.23	11.99	**−8.2900**	−19.5000	−29.4800	−39.8800	−20.5201	−18.6072
Sub22	35.35	42.40	56.14	78.61	27.81	**24.04**	**−14.9700**	−30.7500	−34.1500	−49.6300	−24.8762	−16.1279
Sub28	**24.00**	27.59	38.69	50.44	31.65	29.85	**−11.1100**	−23.9100	−34.7100	−49.3800	−33.0837	−24.7822
Sub37	11.40	12.81	19.91	30.92	12.58	**10.29**	**−8.6100**	−10.7400	−23.0900	−38.5600	−15.2173	−11.4828
Sub46	**15.64**	16.35	20.37	29.00	20.51	18.45	**−9.9400**	−11.4000	−20.1400	−39.1700	−29.1230	−23.4824
Sub38	**8.39**	9.70	16.16	22.75	11.52	9.19	**−8.8200**	−12.2500	−30.8000	−43.8900	−16.1222	−12.8955
Sub07	**11.19**	12.58	17.41	26.57	13.61	11.72	**−9.5600**	−13.8200	−20.1000	−31.2800	−14.1214	−12.2019
Sub10	**22.68**	24.80	31.32	40.73	29.31	27.65	**−11.4600**	−12.8500	−26.7300	−44.3100	−22.8050	−18.6480
Sub03	**27.37**	29.00	48.31	59.93	32.37	30.70	**−13.3200**	−20.7800	−36.3800	−48.7000	−32.0430	−23.0302
Sub36	28.77	32.15	48.72	59.42	25.46	**25.12**	**−14.4100**	−22.4000	−35.4200	−49.5400	−38.9763	−34.9449
Sub47	11.05	12.49	20.52	27.99	11.58	**9.39**	**−10.6300**	−16.7400	−32.1700	−47.9000	−16.8812	−13.0657
Sub25	34.66	40.41	43.66	57.35	**34.34**	34.49	**−20.1100**	−30.1500	−34.1500	−49.5900	−27.4416	−21.8431
Sub30	**17.89**	18.87	23.13	28.76	22.45	20.73	**−10.8100**	−14.4100	−20.5700	−36.3200	−23.4442	−18.7374
Sub45	16.32	19.63	28.13	33.65	17.01	**15.23**	**−9.7000**	−22.2900	−33.9200	−48.4600	−24.7264	−18.8631
Sub39	**7.86**	14.21	23.21	31.00	17.55	16.37	**−7.5700**	−18.2400	−35.6900	−46.8700	−38.2363	−33.1955
Sub41	**25.22**	28.51	32.37	46.46	30.27	28.09	**−15.5400**	−27.8500	−32.6700	−48.7900	−29.8998	−21.6876
Sub35	**13.32**	16.15	28.32	34.36	16.86	14.93	**−9.8700**	−20.7600	−35.6700	−48.8100	−26.4565	−19.5712
Sub17	**7.07**	8.41	12.92	16.62	10.88	9.64	**−7.9100**	−10.9400	−20.5400	−27.1500	−15.9567	−13.6174
Sub32	**12.57**	18.38	23.39	33.40	16.89	15.17	**−10.3900**	−21.6900	−33.4600	−44.9900	−17.8512	−15.0290
Sub23	**16.22**	16.49	19.90	26.44	18.93	17.91	**−15.2500**	−16.8900	−22.5300	−36.4900	−20.7057	−19.2157
Sub18	17.96	19.97	24.78	30.58	18.44	**17.42**	**−10.7300**	−17.8500	−26.3900	−38.5900	−14.6950	−14.3062
Sub13	**9.72**	14.14	28.85	39.03	18.01	14.70	**−9.9800**	−19.5200	−35.8100	−49.0600	−27.8731	−17.9618
Sub05	24.80	27.29	38.17	45.63	26.14	**24.69**	**−12.2000**	−22.1600	−32.9400	−46.4000	−22.2265	−16.9284
Sub44	**7.76**	11.61	11.40	17.80	15.16	12.92	**−7.7900**	−17.2000	−23.1400	−36.0500	−27.3538	−21.6972
Sub01	26.72	28.18	35.08	46.16	26.75	**24.94**	**−11.7400**	−19.3100	−33.6500	−49.1600	−20.5735	−16.8944
**Mean**	17.65	20.58	28.25	37.39	20.77	19.02	−11.2284	−18.9760	−29.7720	−43.5588	−24.0484	−19.1527

**Table 5:** Compact repeated-results comparison across subject-aligned metrics with six methods. Values are reported as mean ± standard deviation, and the last column reports *p* values from two-sided Wilcoxon signed-rank tests comparing Ours and Scratch on paired subject-level outcomes.

Metric	Ours	Scr	Rule	PD-B	FeatAlign	Zero	*p* (Ours vs. Scr)
MSE Hip	4.0143 ± 1.3136	5.9544 ± 2.4457	8.3693 ± 5.1398	9.6776 ± 4.7966	9.4481 ± 4.8333	11.5103 ± 6.9183	$7.51\times 10^{-8}$
MSE Knee	5.4507 ± 1.7431	6.5507 ± 2.6541	6.9683 ± 3.4544	6.2857 ± 2.1345	5.7793 ± 2.0833	10.7025 ± 4.1805	$1.51\times 10^{-5}$
MSE Ankle	2.4719 ± 1.7180	2.3630 ± 1.3550	5.9244 ± 4.7532	4.2786 ± 2.0278	4.1340 ± 1.9635	9.1827 ± 6.5866	0.9578
Total MSE	11.9369 ± 3.4081	14.8681 ± 4.8668	21.2621 ± 9.6127	20.2420 ± 6.6225	19.3614 ± 6.5181	31.3955 ± 13.3157	$5.96\times 10^{-8}$
Reward	−18.4798 ± 7.0654	−21.2264 ± 5.5398	−28.3779 ± 7.3077	−24.3079 ± 7.7734	−21.8160 ± 7.1105	−41.4144 ± 7.2752	$3.76\times 10^{-4}$
Jerk	0.0051 ± 0.0117	0.0069 ± 0.0155	0.0009 ± 0.0005	0.0027 ± 0.0021	0.0019 ± 0.0020	0.0004 ± 0.0002	$4.54\times 10^{-5}$

**Table 6:** Ablation study of NIPA, pretraining, and freezing strategy. Values are reported as mean ± standard deviation across repeated evaluations.

Metric	No Pretraining	Random + Full FT	Random + Frozen FE	NIPA + Full FT	NIPA + Frozen FE
NIPA	No	No	No	Yes	Yes
Pretraining	No	Yes	Yes	Yes	Yes
Freezing	No	No	Yes	No	Yes
MSE Hip	5.9544 ± 2.4457	5.1050 ± 2.0716	6.3523 ± 2.2888	5.5257 ± 1.4637	4.0143 ± 1.3136
MSE Knee	6.5507 ± 2.6541	5.5735 ± 2.4782	5.6449 ± 2.4408	4.6894 ± 0.2408	5.4507 ± 1.7431
MSE Ankle	2.3630 ± 1.3550	3.6280 ± 0.6404	3.6405 ± 0.6600	2.6312 ± 0.6702	2.4719 ± 1.7180
Total MSE	14.8681 ± 4.8668	14.3065 ± 3.2659	15.6376 ± 3.4189	12.8463 ± 1.7676	11.9369 ± 3.4081
Reward	−21.2264 ± 5.5398	−21.4454 ± 6.6561	−20.5479 ± 6.5645	−20.3965 ± 2.1160	−18.4798 ± 7.0654
Jerk	0.0069 ± 0.0155	0.0317 ± 0.0246	0.0117 ± 0.0108	0.0305 ± 0.0464	0.0051 ± 0.0117

## Data Availability

The data presented in this study were derived from public domain resources. The clinical dataset analyzed in this study is publicly available from Van Criekinge et al. at https://doi.org/10.1038/s41597-023-02767-y. Derived data and custom code supporting the findings of this study are available from the corresponding author on reasonable request.
